# Distal renal tubular acidosis in primary Sjögren syndrome

**DOI:** 10.1186/1479-5876-10-S3-P8

**Published:** 2012-11-28

**Authors:** Tim Both, Ewout J Hoorn, Zana Brkic, Marjan A Versnel, Jan AM van Laar, P Martin van Hagen, Robert Zietse, Paul LA van Daele

**Affiliations:** 1 Dept. of Internal Medicine, Erasmus MC, Rotterdam, The Netherlands; 2 Dept. of Immunology, Erasmus MC, Rotterdam, The Netherlands

## Introduction

Primary Sjögren syndrome (pSS) is a chronic inflammatory disorder characterized by lymphocytic infiltration of exocrine glands. pSS can also cause distal renal tubular acidosis (dRTA). dRTA is a disorder in which patients are unable to acidify their urine because of impaired hydrogen ion secretion in the collecting duct.

## Aim

To determine the prevalence of dRTA in pSS using a urinary acidification test.

## Patients and methods

62 pSS patients and 27 healthy controls participated in the study. After baseline measurements, both groups received a single administration of 40 mg furosemide and 1 mg fludrocortisone after which urine pH was measured hourly for six hours (Walsh *et al.*, Kidney Int 2007). dRTA was initially defined as a failure to achieve a urine pH < 5.3.

## Results

At baseline, pSS patients had a significantly higher urine pH (6.2 ± 0.6 vs. 5.8 ± 0.7) and lower estimated ammonium secretion (10 ± 14 vs. 25 ± 23 mmol/l) than controls (p < 0.01 for both), already suggesting a subtle acidification defect in pSS. Only 4 pSS patients, however, had overt metabolic acidosis (serum bicarbonate < 21 mmol/l). During the test, 24 pSS patients (39%) failed to acidify their urine to a pH < 5.3.

Seven controls (26%), however, were also unable to reach a urine pH < 5.3 (p = 0.3). Therefore, we believe a urine pH of 5.3 may not be sufficiently specific for diagnosing dRTA in pSS. All controls did reach a urine pH of 5.8 or lower during the test. Setting the threshold at this level, 7 patients with pSS (11%) were diagnosed with dRTA.

## Conclusions

The prevalence of dRTA in pSS is relatively high. A urinary acidification test is more sensitive to diagnose dRTA in pSS than serum bicarbonate, but the threshold for a positive test should be set at a urine pH of 5.8 instead of 5.3.

